# Political priority and pathways to scale-up of childhood cancer care in five nations

**DOI:** 10.1371/journal.pone.0221292

**Published:** 2019-08-19

**Authors:** Avram E. Denburg, Adriana Ramirez, Suresh Pavuluri, Erin McCann, Shivani Shah, Tricia Alcasabas, Federico Antillon, Ramandeep Arora, Soad Fuentes-Alabi, Lorna Renner, Catherine Lam, Paola Friedrich, Brandon Maser, Lisa Force, Carlos Rodriguez Galindo, Rifat Atun

**Affiliations:** 1 Division of Haematology/Oncology, The Hospital for Sick Children, Toronto, Canada; 2 Child Health Evaluative Sciences, Peter Gilgan Centre for Research and Learning, The Hospital for Sick Children, Toronto, Canada; 3 Institute of Health Policy, Management and Evaluation, Dalla Lana School of Public Health, University of Toronto, Toronto, Canada; 4 Harvard School of Public Health, Harvard University, Boston, Massachusetts, United States of America; 5 Philippine General Hospital, Manila, Philippines; 6 School of Medicine, Universidad Franciso Marroquin, Guatemala City, Guatemala; 7 Unidad Nacional de Oncología Pediátrica, Guatemala City, Guatemala; 8 Max Super Specialty Hospital, New Delhi, India; 9 Hospital Nacional de Niños Benjamin Bloom, San Salvador, El Salvador; 10 Korle Bu Teaching Hospital, Accra, Ghana; 11 Department of Global Pediatric Medicine, St. Jude Children’s Research Hospital, Memphis, Tennessee, United States of America; Institute of Economic Growth, INDIA

## Abstract

**Background:**

Despite increasing global attention to non-communicable diseases (NCDs) and their incorporation into universal health coverage (UHC), the factors that determine whether and how NCDs are prioritized in national health agendas and integrated into health systems remain poorly understood. Childhood cancer is a leading non-communicable cause of death in children aged 0–14 years worldwide. We investigated the political, social, and economic factors that influence health system priority-setting on childhood cancer care in a range of low- and middle-income countries (LMIC).

**Methods and findings:**

Based on in-depth qualitative case studies, we analyzed the determinants of priority-setting for childhood cancer care in El Salvador, Guatemala, Ghana, India, and the Philippines using a conceptual framework that considers four principal influences on political prioritization: political contexts, actor power, ideas, and issue characteristics. Data for the analysis derived from in-depth interviews (*n* = 68) with key informants involved in or impacted by childhood cancer policies and programs in participating countries, supplemented by published academic literature and available policy documents.

Political priority for childhood cancer varies widely across the countries studied and is most influenced by political context and actor power dynamics. Ghana has placed relatively little national priority on childhood cancer, largely due to competing priorities and a lack of cohesion among stakeholders. In both El Salvador and Guatemala, actor power has played a central role in generating national priority for childhood cancer, where well-organized and -resourced civil society organizations have disrupted legacies of fragmented governance and financing to create priority for childhood cancer care. In India, the role of a uniquely empowered private actor was instrumental in creating political priority and establishing sustained channels of financing for childhood cancer care. In the Philippines, the childhood cancer community has capitalized on a window of opportunity to expand access and reduce disparities in childhood cancer care through the political prioritization of UHC and NCDs in current health system reforms.

**Conclusions:**

The importance of key health system actors in determining the relative political priority for childhood cancer in the countries studied points to actor power as a critical enabler of prioritization in other LMIC. Responsiveness to political contexts–in particular, rhetorical and policy priority placed on NCDs and UHC–will be crucial to efforts to place childhood cancer firmly on national health agendas. National governments must be convinced of the potential for foundational health system strengthening through attention to childhood cancer care, and the presence and capability of networked actors primed to amplify public sector investments and catalyze change on the ground.

## Background

Despite increasing global attention to non-communicable diseases (NCDs) and their incorporation into universal health coverage (UHC), the factors that determine whether and how NCDs are prioritized in national health agendas and integrated into health systems remain poorly understood. Childhood cancer is a leading non-communicable cause of death in children aged 0–14 years worldwide [1,2]. More than 80% of diagnosed cases of childhood cancer occur in low- and middle-income countries (LMIC) [3], where access to diagnostics and treatment are limited [4]. The substantial improvements in pediatric cancer survival in high-income countries (HIC) in the last five decades have not been realized in LMIC [5]. Although locally-led endeavors, including ‘twinning’ partnerships between HIC and LMIC institutions [6], have tried to address these disparities, childhood cancer care is not incorporated into universal health coverage (UHC) in many LMIC, nor integrated into broader systems of care. Consequently, these initiatives have not reached scale, resulting in limited impact on survival and mortality at the population level [7,8].

Scale-up of effective and sustainable childhood cancer services and their incorporation within UHC expansion in LMIC requires attention to political, social, and health system contexts [9,10]. Improved knowledge of how childhood cancer programs are introduced and scaled in health systems in LMIC is key to both sustainable improvements in childhood cancer services across the care continuum and broader health system strengthening. In introducing UHC and setting priorities, policymakers in LMIC face difficult choices in the allocation of scarce resources to competing health needs. The generation of priorities is a complex and often fraught process, determined by multiple interrelated factors [11,12]. Absent an understanding of this process, key opportunities to incorporate childhood cancer in UHC and integrate programs of care in health systems will be missed.

Based on in-depth qualitative case studies, we analyze the determinants of priority setting for childhood cancer care in a range of LMIC selected from different geographical regions and varied stages of cancer care development. Our findings provide insights into key barriers and enablers related to prioritizing childhood cancer care, its incorporation in UHC, and its integration in health systems, and yield lessons for countries scaling up childhood cancer care in the context of efforts to expand UHC and meet Sustainable Development Goal (SDG) 3 targets [13].

## Methods

### Study settings

Our selection of case studies sought to balance geographical range, political organization, health system development, and project feasibility (Table 1). We aimed to incorporate LMIC with varied childhood cancer outcomes stages of childhood cancer policy and program development. From a cross-section of potential comparators, our sample was further refined based on the strength and reliability of investigator relationships with local research partners and professional networks. The comparator countries–El Salvador, Guatemala, Ghana, India, and the Philippines–represent different geographic regions, cultural backgrounds, macroeconomic realities, and political traditions. Our analysis strove to situate and understand health system priority-setting in light of these varied contextual factors. We included two countries with shared regional realities, El Salvador and Guatemala, to retain a measure of commonality amidst diversity that might set in relief key differences responsible for variations in the national political priority for childhood cancer.

**Table 1:** Key economic, health, and childhood cancer indicators in the case countries.

**Indicator**	**El Salvador**	**Guatemala**	**Philippines**	**India**	**Ghana**
Population in millions / Pediatric population aged 0–14 (% of total)	6.3/26.4	16.6/36.2	103.3/32	1324/28.2	28.2/38.7
Population living below national poverty line (%)	31.8	59.3	21.6	21.9	24.2
Population living below the international poverty line (%)^a^	1.9	8.7	7.8	21.2	13.3
Average life expectancy for males/females (years)	68.6/77.7	68.5/75.6	65.8/72.7	67.1/70.2	61.7/63.7
GINI index	40	48.3	40.6	35.7	43.5
Infant mortality per 1,000 live births	14	24	21.5	33.6	37.2
Maternal mortality per 100,000 live births	54	88	114	170	319
GDP/capita (current International $)	8316	8447	8935	7762	4738
Total health expenditure (% GDP)	6.96	5.82	4.39	3.66	4.45
Total health expenditure per capita (current Int $)	599.5	462.4	342.3	241.5	189.4
Estimated total incident cancer cases in 2015, ages 0–14 (95% UIs)[14]	359 (248–489)	1366 (983–1829)	5709 (4830–6929)	70615 (61766–80388)	4720 (3412–6614)
Estimated 5-year survival (%), pediatric acute lymphoblastic leukemia (95% UIs)[14]	41 (9–76)	41 (9–76)	39 (10–65)	58 (40–78)	24 (5–58)
Total active pediatric oncologists^b^ / Incident cases per oncologist	3/120	9/152	49/117	150/471	3/1573

Drawn from the World Bank Group Development Indicators, 2014–2016 data

^a^ $1.90 per day, 2011 PPP (purchasing power parity)

^b^ Expert estimates

### Conceptual framework

Political priority is established through explicit recognition of a problem by political leaders, the enactment of policies designed to address the problem, and the corresponding allocation of resources to support their implementation. To analyze the determinants of childhood cancer prioritization and policy development in the countries studied, we apply an established conceptual framework by Shiffman and Shah that has been used to analyze factors influencing political prioritization of a range of health issues at both national and global levels of governance, including maternal and neonatal health, child development, and surgical care [15, 16, 17, 18]. It considers four principal influences on priority-setting: (1) political contexts, (2) actor power, (3) ideas, and (4) issue characteristics (S1 Table)[19]. We employed this framework to balance clarity, when comparing a diverse range of health system contexts, with explanatory power, through incorporation of key domains common to a number of prevailing policy analytic frameworks. Our findings are based on literature review and in-depth interviews with key informants, guided by the Pediatric Oncology System Integration Tool (POSIT), an expert-informed, peer-reviewed instrument for analyzing childhood cancer in health system context [20]. Focal domains of analysis included: the place of childhood cancer within the broader health system and policy environment; planning and priority setting processes for childhood cancer care; and modalities of resource generation and distribution for childhood cancer programs and services.

### Data collection

The study employed a multiple case study design [21] that emphasized policy decision-making at the national and facility levels, with attention to the institutions, actors, and processes that mediate policy and program development. Data for the analysis derived from: (1) structured searches of the published and grey literature on the health system context and childhood cancer care in participating jurisdictions, including academic articles, governmental and non-governmental documents, media sources, and organizational and industry websites; and (2) in-depth, semi-structured interviews with key informants involved in or impacted by childhood cancer policies, programs or services in participating countries. Drawing on POSIT and the analytic framework developed by Shiffman and Shah, we developed a semi-structured interview guide focused on the governance and financing of childhood cancer care (S1 Fig). Between February 15 and September 1 2017, we interviewed a stratified purposive sample of key informants (*n* = 68: El Salvador = 19; Guatemala = 13; India = 14; Philippines = 12; Ghana = 10) representing governmental, health care, and advocacy roles instrumental to policy processes and program development on childhood cancer in participating jurisdictions (S2 Table). We interviewed informants at all major administrative levels of the health system, from community and district positions to regional and national ones. Participants ranged in seniority, representing early-career (1–5 years; *n* = 18), mid-career (6–15 years; *n* = 29), and senior (16+ years; *n* = 21) levels of experience in their respective fields. Participants were identified through grey literature review, scans of relevant governmental and institutional websites, and referral by local study team members or prior interviewees, and were recruited by email, phone, or in-person through introduction from local collaborators. The size and breadth of the sample of interviewees was determined through constant comparison with existing themes as the analysis of interviews proceeded [22].

### Data analysis

Literature searches followed a scoping review approach [23,24]. Qualitative interviews were audiotaped, transcribed verbatim, and translated into English where relevant. Relevant literature and interview transcripts were imported into and inductively coded using NVivo 11 software (QSR International, Ltd.). Independent coding of each interview was completed by one of four authors (AR, SP, SS, EA). Team workshops were held to iteratively review and compare coding systems. Random samples of the data from each country were double-coded to ensure broad consistency in approach. Drawing on a constructivist grounded theory approach, the data underwent sequential phases of coding, moving from open through theoretical codes, with constant comparative methods employed to refine codes, establish analytic distinctions, and capture emergent themes [25]. Additional interviews were conducted as needed to pursue relevant themes as they emerged, until theoretical saturation was achieved. We employed the major domains (political contexts, actor power, ideas, and issue characteristics) from Shiffman and Shah’s framework for political prioritization as sensitizing concepts to organize and guide our analysis [26]. Local investigators in each country constructively reviewed the manuscript to maximize the fidelity and reliability of our findings in country context.

### Ethics

We obtained institutional review board approval from the Harvard T.H. Chan School of Public Health IRB, and study exemption from institutions in other participating jurisdictions, including St. Jude Children’s Research Hospital, Korle Bu Teaching Hospital, Hospital Nacional de Niños Benjamin Bloom, Tata Memorial Centre, and Unidad Nacional de Oncologia Pediatrica. Written informed consent was obtained prior to each interview. Participant confidentiality was protected through unique, anonymized identifiers assigned to each interviewee, stored in a delinked and encrypted file.

## Results

### Political contexts

The political, economic and health system contexts in which childhood cancer programs and services operate, and with which policy communities must contend, are critical determinants of the relative prioritization of childhood cancer amongst competing health issues.

#### National governance structures and policy windows

In the countries studied, political stability and commitment were seen as necessary preconditions for the integration of childhood cancer care within health system. However, crucial first steps were taken by philanthropic organizations, sometimes in the face of political instability and competing policy priorities. Capitalizing on the independent progress made by philanthropic organizations has required dedicated energy to generate political momentum for incorporating childhood cancer in the context of broader health system reforms. In a number of the countries, growing national commitment to both UHC and to fight non-communicable diseases (NCDs) has emerged as a unique opportunity to influence the relative priority for childhood cancer.

The momentum for UHC in El Salvador is seen by many as a “policy window” for the prioritization and incorporation of pediatric cancer care in the basket of publicly covered services (PRA4-ES, S2-ES, S3-ES) [27]. This push for health system strengthening and financial risk protection is relatively new, and contrasts with a history of weak public sector stewardship of health care [28]. The initial stages of pediatric cancer care development took root in, and were shaped by, this political backdrop. In the context of a political climate that limited public health system investment but encouraged external and private investment in health, an alliance between the Hospital Nacional de Ninos Benjamin Bloom (HNNBB), St. Jude Children’s Research Hospital (SJCRH), and the private non-profit Fundacion Ayudame a Vivir was forged to create a pediatric cancer program in El Salvador in 1993 [29].

“We’ve gone through seven different governments in 24 years and we have never had an issue. If you ask me what was one of the keys for this program to reach…the success that it’s had, it is thanks to the collaboration and commitment between these three entities.” (PRA1-ES)

To expand its services and accommodate larger patient volumes, the outpatient Centro Medico Ayudame a Vivir opened in 2008 on adjacent land donated by the national government. The program continues to operate as a well-integrated public-private partnership (PRA1-ES, NGO1-ES). The bulk of the care is delivered within HNNBB, a publicly-funded government hospital, but it is financed heavily by philanthropy and revenues funneled through the Fundacion. Just as importantly, the Fundacion’s Board of Directors serves as a principal agenda-setting body for the national childhood cancer program, deliberating on major policy, administrative, and financial decisions (NGO1-ES, PRA5-ES, NGO5-ES).

While the centrality of this public-private partnership to childhood cancer care in the country persists, the increasing commitment of the government to UHC-oriented health system reforms has created opportunities to further integrate pediatric cancer programs and services into public sector priorities (PRA2-ES) [30]. Recent government-led reforms, articulated in its *Plan Quinquenal de Desarrollo 2009–2014*, have focused on primary care strengthening and the extension of health coverage to broader swaths of the populace, with an emphasis on the most vulnerable [31]. Investment in family health teams (Equipos de Salude Familiar, ECOS) has empowered local municipalities to increase participation and ownership in local health-related initiatives [32].

“Before…we didn’t know what was happening. Now, the healthcare model begins in the community. The healthcare system looks to the community for cases that could require a larger amount of help, but it begins with ECOS [Equipos de Salude Familiar]. They establish priorities as to what they want the government to do…. That’s when they put emphasis on illnesses, non-communicable diseases, and that’s where pediatric cancer comes in.” (S2-ES)

Attention to improved coordination amongst public sector institutions and service providers, and the development of a unified health information management system, have encouraged the formation of an integrated network of public health services (S3-ES, S4-ES) [33,34]. In this environment, key points of integration between the existing childhood cancer program and the broader health system have cemented. ECOS now function as an essential node for early cancer detection and referral to HNNBB (PRA4-ES, S2-ES). The childhood cancer program in turn leverages ECOS capacities for psychosocial and palliative care to reduce barriers to treatment abandonment, monitor and support medication compliance, and deliver elements of supportive care closer to home (S5-ES) [27, 30].

“When the parents want the child to pass away at home, the ECOS visit them at home. We communicate with them… to understand whether they are giving the medication appropriately and… according to the indications for which they were prescribed. And how do we make sure that the patient is alive? (It is) through the ECOS. The healthcare system is double checking, it is informing us.” (S4-ES)

Even so, the fit of governmental priorities with childhood cancer needs in El Salvador remains imperfect. A notable example is the substance of national NCD and cancer-specific commitments. Together, the 2010–2014 National Strategic Program for the Promotion of Health: Prevention and Control of Non-Communicable Chronic Diseases, and the 2015 National Policy on Integrative Care for People with Cancer (NCP), represent the first formal inclusion of cancer amidst national health system priorities [35, 36]. Neither, however, deal substantively with childhood cancer. The NCP’s emphasis on cancer screening and prevention is poorly aligned with childhood cancer program needs. Consequently, stakeholders in the childhood cancer community view the NCP as of minimal relevance to the national childhood cancer program, and worry about divergence between evolving national cancer priorities and the needs of children with cancer (PRA3-ES, S1-ES).

“They wanted to group cancer, children and everybody together, into one big monster. I mean, it’s great that the government wants to take care of the $3 million per year that it costs to maintain this program. Let’s hope that when it happens…I mean, I have a lot of doubt they’re going to continue with the same quality of treatment.” (NGO1-ES)

In Guatemala, by contrast, a lack of any sustained overarching priority placed on NCDs has prevented a comparable policy window from opening. The government continues to struggle to provide basic health services to its population, including for the control of prevalent communicable diseases (S2-GUA). It has a poorly defined health system strategic plan, and is not engaged in sustained discussions to develop a meaningful NCD or cancer program [37, 38]. As a direct extension, the priority placed childhood cancer care at the national level remains minimal.

“It’s a list of good intentions basically. It’s not a plan per se. It’s a proposal to make improvements. It’s a plan that’s very general that doesn’t conclude with how you’re going to operationalize it. It doesn’t assign any resources to put this plan into practice. It’s a proposal of actions that should be taken.”(PRA3-GUA)

In the resultant political vacuum, a unique public-private partnership has been instrumental in advancing childhood cancer care capacities in Guatemala. Unidad Nacional de Oncologia Pediatrica (UNOP), a public referral hospital for pediatric cancer cases, was formed in 2000 as a multi-institutional collaboration between Guatemala’s Ministerio de Salud Publica y Asistencia Social (MSPAS), SJCRH in the United States (US), and a private, non-profit local foundation called Fundacion Ayudame a Vivir Guatemala (AYUVI) [39]. UNOP operates a stand-alone facility containing inpatient and outpatient childhood cancer services; it also houses AYUVI’s administrative offices. Despite its proximity to Roosevelt Hospital, a national referral hospital for children and adults, UNOP has developed internal capacities for most core elements of care, including laboratory, blood bank, radiology, intensive care, and emergency services (S1-GUA). External consultations are contracted with specialized providers and centers, both public and private, in exchange for monetary reimbursement.

“Cancer, you either do it properly or children die…What we have learned is that we’re going to have to get out of the National Healthcare System. We had to be independent because, depending on other centers that don’t have the capacity to give us what we need, that affects the child’s survival. So, say, I want radiotherapy, certain images, blood banks, we have to decide to pay for it…through the private system.” (S2-GUA)

Policy agenda-setting and development for childhood cancer fall largely to the Medical Directorate of UNOP. Though MSPAS and AYUVI are formally represented at board meetings, policy direction issues mainly from UNOP itself, rather than the Ministry (S1-GUA, PRA3-GUA).

Due in part to these unique governance structures, national reach remains a challenge for childhood cancer care in Guatemala. UNOP’s estimated population coverage of expected incident childhood cancer cases is 47 percent (S1-GUA, NGO1-GUA, PRA1-G UA). It struggles to reach remote or mountainous regions that have poor infrastructure, high poverty rates, and a reliance of traditional healing methods. As a result, roughly 30 percent of patients present with evidence of metastatic disease, of which a disproportionate percentage are of indigenous origin (PRA3-GUA, S1-GUA). To improve its referral basis, UNOP has forged relationships with specific hospitals to assume care of new pediatric cancer cases.

“We are a national referral hospital, which means that any hospital can refer us patients if they suspect that there might be cancer in the child. And we’ve received all of them. And once the diagnostic is complete, we can serve patients or we can confirm the illness, and… We refer them to the proper channel. So, there is that relationship with the rest of the services, public and private, is that they have the ability to refer patients.” (PRA3-GUA)

UNOP has also placed an emphasis on human resource development, and serves as a training site for a range of pediatric fellowships (S1-GUA, S2-GUA). In an effort to provide care closer to its patients–to ‘deconcentrate rather than decentralize’, in the words of one stakeholder–UNOP operates an outpatient treatment center in Xela, a region where close to 40 percent of its patients originate (PRA3-GUA, NGO1-GUA). Nevertheless, health system integration of childhood cancer services across the care continuum remains a challenge in Guatemala. The absence of robust and sustained political attention placed on NCDs or UHC at the national level has barred windows of opportunity for the integration of childhood cancer care in broader health system priorities.

Childhood cancer policy development in the Philippines shares features of both the El Salvadorean and Guatemalan experiences, but is distinguished by a political context conducive to greater ‘top-down’ momentum for system reform. Historically, the country has faced health system governance challenges due to its extensive geography with many island communities and the service decentralization [40]. More than 30 centers provide care for children with cancer in various capacities across the country: many of these are insufficiently equipped to deliver high-quality care; only three of these institutions have the designated units and multidisciplinary teams necessary for comprehensive pediatric cancer care (PRA1-PHI, HI1-PHI) [41,42]. This system fragmentation complicates coordination across primary, secondary and tertiary levels of care.

However, a recent strong centralized push to both expand UHC and improve NCD care is helping to ameliorate these governance challenges (PRA1-PHI, HI4-PHI, S2-PHI). In alignment with UHC-centered health system financing reforms, which we explore in detail below, the government has broadened the purview of the Philippine Cancer Control Program (PCCP) to include childhood cancer (PRA1-PHI, HI1-PHI). One regulatory authority emphasized the perceived need for dedicated policy attention to childhood cancer: “*It cannot be cancers in general*, *there has to be specific language for children”* (PRA4-PHI). An initial focus on acute lymphoblastic leukemia has integrated expanded insurance coverage and enhanced support for diagnostic and treatment capacities to improve access to care for children with the disease (S2-PHI, HI2-PHI) [43]. A bill sponsored by the Department of Health (DOH), entitled the National Integrated Cancer Control Act (NICCA), proposes further expansions in coverage and service delivery for children with cancer [44]. Optimism regarding its passage–in contradistinction to its failed predecessor–is high, in large measure due to a shift in governmental priorities from communicable to non-communicable diseases in the intervening years (PRA1-PHI, HI1-PHI). In parallel, the DOH is developing plans to build and designate specific comprehensive cancer centers across the Philippines, with a goal of providing access to quality care at hospitals outside urban hubs. These centers will be required to provide comprehensive services in accordance with specific clinical guidelines and accountability requirements (PRA1-PHI, HI1-PHI). An added emphasis on health system responsiveness to patient journeys through the care continuum is facilitating increased networking and collaboration across health care facilities and tiers (S1-PHI, E1-PHI) [45].

In India, by contrast, the Ministry of Health and Family Welfare (MOHFW) has placed relatively little emphasis on childhood cancer, either through its child health initiatives or NCD strategies–be it the National Cancer Control Program (NCCP) or the umbrella National Program for Prevention and Control of Cancers, Diabetes, Cardiovascular Diseases, and Stroke (NPCDCS) (S5-IND, PRA4-IND) [46]. In the absence of strong central leadership on childhood cancer through the MOHFW, unique governance structures evolved to attend to the growing burden of childhood cancer.

Tata Memorial Center (TMC) in Maharashtra state is a prominent example of this. It leveraged powerful private philanthropic origins to secure distinct, dedicated channels of public funding and accountability, and to embed itself in the health system as a national referral center for cancer, including cancers in children (S2-IND, PRA1-IND). Established in 1941 by the Sir Dorabji Tata Trust, responsibility for the management and operation of Tata Memorial Hospital was handed over to the MOHFW in 1957, and subsequently, along with its Cancer Research Institute, to the Department of Atomic Energy (DAE) in 1962, making it the only medical facility under the aegis of the DAE. The resultant governance model has allowed TMC to circumvent many of the resource challenges faced by government-administered hospitals. Along with greater degrees of financial autonomy, the position of the DAE directly under the Prime Minister’s Office (PMO) has minimized the bureaucratic inefficiencies associated with other government hospitals. TMC administrators have more direct access to the PMO to petition for budgetary priorities, expand the institution’s service capacities, and introduce new policies or programs on cancer care.

“For various reasons, we feel that it’s important it [the partnership] stays that way, because we are not competing with a thousand other hospitals for funding and for getting the Ministry’s attention.” (S5-IND)

“…the Department of Atomic Energy is managed by the Prime Minister’s Office…So, administration is much easier and there is less bureaucracy in the stuff I do, any of the departmental bodies and how they function. It’s great and, for them, this is the only hospital which is there at the moment in terms of oncology care.” (PRA3-IND)

These unique attributes of governance have made TMC an independent player in policy and program development for childhood cancer in India. By contrast, MOHFW stewardship of cancer care is perceived as relatively haphazard, and out of step with the capacity for innovation, and consequent progress, achieved at TMC. The impact of these asymmetric capacities on the health system reach of TMC’s cancer care policies and programs is uncertain, though it is evident that wide disparities in access to, and quality of, care for children with cancer persist across the country. In effect, this island of excellence has faced systemic difficulties in diffusing its innovations to the encompassing health system, at least in part due to the parallel governance structures that have enabled those innovations.

“The ICMR runs the cancer registry program in the country. TATA now has started running a parallel cancer registry program in the country. There’s no cross-talk between the two registries…so there is a mismatch…TATA is doing eminent work, but it is a mismatch with the Ministry of Health’s program.” (S3-IND)

Health system governance structures in Ghana also create parallel channels of authority, though with less salutary results for national priority-setting on childhood cancer. Public facilities provide roughly half of Ghana’s healthcare services, and are overseen by Ghana Health Services, which functions as the implementation agency of the MOH. Teaching hospitals are excluded from the remit of GHS [47]. This has provided them operational independence, but has also limited their influence in health system priority-setting in at least two ways: firstly, by compromising the inclusion of institutional data in national processes health system monitoring and evaluation, and secondly, by weakening the influence of institutional and program leaders in GHS-led reforms (PRA1-GHA) [48]. This is of crucial significance for organized childhood cancer programs, which are highly centralized and administered through two tertiary-care teaching hospitals.

### Economic environments

Differences in the respective economic environments for health system financing in the country case studies emerged as key factors of childhood cancer care prioritization and system integration. Health system financing in Ghana consists of a mix of public and private modalities, with the majority of total health expenditure derived from government sources. The tax-financed National Health Insurance Fund, which constitutes the lion’s share of public health financing in Ghana, lacks coverage for all treatment and drug costs related to childhood cancer [49]. What little funding does exist for childhood cancer care is provided largely by external donors; there are no formal financing provisions specific to childhood cancer for the Non-Communicable Disease Control Program or National Cancer Plan. However, there is a notable degree of path dependency to health system financing induced by population-level disease burden and international donor priorities, which remain focused on communicable diseases (PRA2-GHA) [45]. Earmarked funds from external donors for priority vertical programs continue to influence allocative priorities within the health system, which have tended to exclude NCDs, and childhood cancer in particular [50].

“For all public health policies, the burden influences such policies. With regards to childhood cancer, we are looking at the burden, which is comparatively low, because we are in an environment where communicable diseases are many, so the burden.” (BMA1-GHA)

“I think we haven’t really had a big look around, what is the prevalence rate of childhood cancers, which childhood cancers can we cover, which ones are too much or too heavy on the insurance…We haven’t discussed that, so I think that has been the biggest issue.” (BMA2-GHA)

In the Philippines, by contrast, a signal health policy achievement for childhood cancer has been the development of government-funded initiatives to pay for the care of children diagnosed with cancer. The PhilHealth insurance corporation has created the ‘Z Benefits’ program, which provides comprehensive coverage to children diagnosed with ALL and prioritizes ‘service patients’ otherwise unable to pay the high out-of-pocket costs of care (PRA1-PHI, S2-PHI, HI3-PHI) [38]. The ALL Medicine Access Program operates in conjunction with the Z Benefit to provide free chemotherapy for patients diagnosed with ALL [41]. The government has also created a ‘no-balance billing’ system for service patients that requires hospitals to absorb any additional costs for the patients receiving the Z Benefit (PRA1-PHI, H1-PHI) [51]. This program was created in the context of the recent government prioritization of, and commitments to, UHC and financial risk protection for all Filipinos.

Still, problems persist and financial barriers to childhood cancer care endure in the Philippines. Despite the creation of a dedicated modality of financing for childhood cancer, the diffuse and fractured nature of health system financing produces budgetary uncertainty, limiting the population-level impact of financial coverage for children with cancer. Public hospitals receive their major and most consistent funds from several different departments within the government, which are allocated to the DOH and then divided across institutions and programs (HI1-PHI, PRA3-PHI, S6-PHI). PhilHealth reimbursements and individual hospital revenues also contribute to the budget. For cancer initiatives specifically, the Z Benefit and the ALL Medicine Access Program have provided more consistent funding for the most common cancer affecting children. However, if complications arise during treatment, the funding from insurance often falls short (PRA2-PHI, S3-PHI). Large private institutions such as major banks and philanthropic foundations contribute the remaining and somewhat fluctuating portion of the budget–likened by one stakeholder to ‘*waiting for the rain*’ (E2-PHI). These budgetary inconsistencies are compounded by limited physician compensation in the public sector, making it difficult to recruit and retain childhood cancer providers (S4-PHI). The combined effect of variable sources of financing and a narrow scope of coverage is many children with cancer who still face insurmountable financial barriers to accessing essential components of care.

The economic environments in Guatemala and El Salvador, by contrast, are distinguished by the centrality of philanthropic foundations in the generation and distribution of funds for childhood cancer care. Guatemala’s health system is characterized by considerable private sector involvement and spending, including marked out-of-pocket costs; private health insurance (PHI) coverage is limited. The main social health insurance scheme, the Instituto Guatemalteco del Seguro Social (IGSS), extends to only a proportion of the population, resulting in large coverage gaps (S4-GUA). In this context, AYUVI pools and disburses the vast majority of funding for pediatric cancer care, from monies generated through private philanthropic donations and fundraising events (NGO3-GUA). It also administers external funding support from civil society, academia, industry, and global health institutional partners; the proportion of funds derived from external sources has attenuated over time, in line with growth in the foundation’s fundraising capacities and donor pool (PRA2-GUA, NGO2- GUA). The public sector, by contrast, contributes less than a third of UNOP’s operating budget. The breadth of AYUVI’s funding is notable. It supports comprehensive, wrap-around childhood cancer services, ranging from institutional overhead and direct medical costs to the indirect costs of care incurred by families (S4-GUA).

The funding dynamics of childhood cancer care in El Salvador bear similarities to Guatemala, though admit of greater involvement of and coordination with the public sector. As in Guatemala, health care is financed through a mix of public and private modalities: it is marked by constrained and fragmented public budgets, limited reach of both public and private insurance, and resultant gaps in coverage (S4-ES). The national childhood cancer program operating budget is constituted primarily from domestic philanthropic funds administered by the Fundacion Ayudame a Vivir. Direct government allocation to the program represents less than a third of its budget. However, the government and foundation operate a public-private partnership to finance service delivery [52]. The government funds public medical care at HNNBB–including emergency room and inpatient services, operating theatres, and diagnostic services–which constitute essential components of childhood cancer care (PRA4-ES, S2-ES). The foundation finances the operation of the Centro Medico Ayudame a Vivir and covers most cancer-specific costs of care (S4-ES) [50]. Notably, it funds salaries for key HNNBB personnel (all program oncologists and pediatricians, and approximately half of the nursing cohort); covers inpatient chemotherapy and supportive care medications administered at HNNBB; and contributes to capital outlays for high-cost cancer-specific technologies housed at the hospital (PRA5-ES, NGO2-ES). As in Guatemala, private philanthropic funds–generated largely by the Association of Parents and Friends of Children with Cancer (ASAPAC)–provide comprehensive financial coverage for families affected by childhood cancer, including support for social services and the indirect costs of care (S4-ES).

“[ASAPAC] had to be the third arm out of the whole situation, because one is the hospital, the other one is the foundation with the chemotherapy, and then there is ourselves. We are involved in everything that no one else would collaborate in.” (NGO4-ES)

However, the degree of public-private integration in El Salvador has arguably facilitated greater incorporation of childhood cancer care into the public health system than in Guatemala, with positive impacts on program reach and system strengthening (PRA2-ES, PRA6-ES).

Finally, the privileged economic environment in which TMC operates–one distinct from that conditioning either childhood cancer services specifically, or health care generally, in the rest of India–is a foundational reason for its programmatic successes and system leadership. TMC receives the bulk of its government funding directly from the DAE, a stream separate from and unreliant on MOHFW budgets. This has buffered TMC against the vicissitudes of health system financing in the country, allowing the institution to innovate without fear of budgetary shortfalls or institutional insolvency.

“The Ministry of Health has to deal with many, many aspects of health care so obviously their funds get distributed…The Department of Atomic Energy, other than their staff healthcare, their main focus is on cancer so we have a lot of funding from the Department of Atomic Energy. The funding is far better in a model like this…” (S4-IND)

Additional sources of financing for childhood cancer services at TMC derive from patient-specific private and public insurance schemes, corporate donations, and philanthropy from individual donors and non-governmental organizations (S2-IND). TMC’s Improving Pediatric Cancer Care and Treatment (ImPaCCT) Foundation, funded by institutional revenues and private donations, supports access to comprehensive care regardless of socioeconomic background, through activities ranging from defraying medical costs and providing nutritional support for patients, to coordinating free accommodation, vocational training, and psychosocial services for caregivers.

“ImPaCCT Foundation provides holistic support…the treatment refusal and abandonment rates have fallen from 25% to 5%.” (NGO2-IND)

“There are so many families who do not have documents and who are not eligible for government help, or for help from the NGOs, or from the charitable trust. So, then this corpus that we started raising in ImPaCCT Foundation was to help particularly those families who have no documents, but who are very motivated to stay back in Mumbai and take treatment…” (S3-IND)

In effect, TMC’s privileged public funding stream, and the innovation it enabled, created a virtuous circle wherein reputational advantage opened novel channels of funding and further strengthened its economic position. These same dynamics are not at work in most public-sector institutions providing cancer care to children in India. The result is a system governed by not one, but many, economic environments, and typified by extremes in access to care.

### Actor power

The relative power held by individuals and institutions concerned or involved with childhood cancer care is a critical determinant of the national political priority for it. Dynamics ranging from the degree of cohesion inherent in the policy community, the presence of strong leadership therein, the character and influence of signal institutions related to the cause, and the role of civil society in the organization and mobilization of responses to the issue all impact upon the priority for childhood cancer care in a given sociopolitical and health system context. The countries studied evinced different actor power dynamics, with varied degrees of policy community cohesion, individual and institutional leadership, civil society mobilization, and support from external partners. These differences have conditioned variations in the barriers and enablers that shape political priority for childhood cancer.

#### Civil society

The role of civil society in El Salvador and Guatemala is illustrative. In both, private philanthropic foundations have played a central role in political advocacy, resource mobilization, and cross-sectoral partnerships (PRA5-ES, NGO1-ES, NGO2-ES). As discussed above, the Fundacion Ayudame a Vivir in El Salvador and AYUVI in Guatemala have not only helped secure and sustain the predominant channels of funding for childhood cancer care in their respective countries, but have also moved the ‘political needle’ on this issue in both (PRA3-ES, S1-ES, S1-GUA). In concert with key professional leaders from the national childhood cancer programs, the foundations have helped instantiate public-private partnerships to leverage public sector resources and expertise in support of childhood cancer care (S4-ES, PRA3-ES) [31]. In El Salvador in particular, this has begun to translate into positive knock-on effects for the health system more broadly, as capacities for diagnosis and referral across tiers of care strengthen.

Another critical impact of the deep-rooted engagement of civil society in these countries is enhanced public awareness of, and community support for, childhood cancer. El Salvador’s program benefits from robust community involvement, much of which is nurtured by ASAPAC (NGO3-ES) [28]. Achieving buy-in for program support from diverse community groups was a gradual process, one shepherded by ASAPAC and the Fundacion Ayudame a Vivir. As membership in ASAPAC has expanded to include an increasingly diverse social network, awareness of the national childhood cancer program–and the broader issue of childhood cancer–has grown.

“It’s all proceeds from three [corporate] donors and close to 7,000 sponsors who periodically and permanently give us donations…After that, there are never-ending activities. We have one or two marathons per year, we have campaigns, concerts, a whole bunch of different activities.” (NGO1-ES)

“Alone we wouldn’t have been able to help so many people. But now with these local resources, we’re able to receive support, and it worked.” (S4-ES)

Comparably, AYUVI’s success in brand dissemination has not only expanded its donor pool but also helped weave childhood cancer into Guatemala’s national consciousness (PRA1-GUA, NGO1-GUA). Popular countrywide events generate considerable publicity for the cause; indeed, the majority of funds received annually derive from such events.

“This is a project of social responsibility…you have to have this awareness and…it is positioning, making sure that people are aware of the issues.” (NGO2-GUA)

AYUVI has also successfully garnered the support of a range of external stakeholders through international recognition of its commitment to pediatric cancer care and leadership in regional research and professional organizations [53, 54].

“In five years, what was going to take me twenty years in Guatemala…we have in all of Latin America and now we are able to take better decisions and we are able to bring more supports to the children.” (S2-GUA)

In the Philippines, there has been a strong push from patient and family organizations to advocate nationally for more equitable and comprehensive childhood cancer services (PRA5-PHI, NGO1-PHI). The receptivity of government to these voices has progressively increased, with the DOH in some instances soliciting direct feedback from patients and encouraging the public to become active participants in the health system (S1-PHI, E1-PHI).

“Patients are asking for privileges, benefits, rights, that they wished they had when they were going through the process.” (PRA5-PHI, NGO1-PHI)

Community groups and civil society organizations, including Cancer Coalition Philippines, have played an important role in advocacy on the substance and prioritization of key pieces of national cancer legislation, notably NICCA (PRA1-PHI, H1-PHI) [39].

“We are collaborating with various partners and stakeholders to come up with a unified version of the bill…It’s primarily the patients’ organizations. We have the Cancer Coalition Philippines, the multi-specialty societies and the Philippine Cancer Society as well, and the different offices of the Department of Health, includes PhilHealth…almost all stakeholders” (PRA1-PHI).

As compared with El Salvador and Guatemala, however, the respective roles of Filipino civil society and government in health policy development have remained within their traditional spheres, and the degree of integration between them in this regard is limited–an evolutionary product of vastly different political contexts.

Civil society appears to have played a more bounded role in the development of Indian policies and programs for childhood cancer care, and remains embryonic in the Ghanaian context. The role of the Tata Trust as a prime mover in the origin, growth and ascendancy of TMC in childhood cancer care in India is difficult to overstate. While it continues to act as a financial and political steward of the cancer system in the country, policy leadership has largely transitioned to government. The challenge of diffusing TMC’s innovations to the broader health system remains; in India’s complex political environment, civil society is likely to play a less determinant role than it has in El Salvador and Guatemala (PRA3-IND, S1-IND). In Ghana, whose health system is not saddled with the historical and institutional complexities of India’s, a gulf remains between the potential and actual impact of civil society in the childhood cancer space–a function primarily of resource scarcity and competing health sector priorities. Domestic civil society institutions remain under-resourced and disempowered; international NGOs remain focused on communicable disease control, primary care strengthening, and basic maternal and child health services (NGO2-GHA, BMA1-GHA). World Child Cancer is a notable exception: it has partnered with key domestic leaders on childhood cancer to raise awareness, support political advocacy, and strengthen basic health system capacities to improve access to care. Its impact, however, is bounded by governance structures and an economic environment that, at present, constrain enhanced political priority for childhood cancer (NGO1-GHA).

#### Policy community cohesion

Policy community cohesion has also served as a determinant influence of program development and ultimate priority-setting on childhood cancer in El Salvador, Guatemala and the Philippines. While varied in degree, the coalescence of a network on childhood cancer in each country has spanned institutions and sectors, and involved both internal and external actors (PRA4-ES, NGO2-ES, S1-GUA, S1-PHI, E1-PHI) [37, 43]. As discussed above, the Fundacion Ayudame a Vivir in El Salvador has functioned not only as a principal financier of the national childhood cancer program, but also as a nidus for policy agenda-setting and development. Importantly, it has formally linked the public and private sectors in this regard (S4-ES, PRA3-ES). Key relationships with important community stakeholders and government members within the legislative branch, who hold designated seats on the Fundacion’s Board of Directors, have provided the program with a political voice and strong base of support from its inception.

“The board, on a strategic level, the board has always maintained a very good relationship with the government that is in power at the time in the country, whether it’s right or left, and I believe that this has generated the success of our project.” (PRA4-ES)

ASAPAC’s role in formalizing the base of community support has put a broad and empowered public behind the program, which has intensified advocacy for childhood cancer at a national level.

“We know of cases where, in a small town far away, there is a child who had leukemia and no resources to transfer him to the Bloom Hospital. We contact the Mayor, Women’s organizations, or organizations that protect children, and this child, with the Department of Health, we help them to be better taken care of.” (PRA2-ES)

In Guatemala, comparable partnerships across the public and private sectors have engendered a network of diverse stakeholders mobilized around a common purpose. UNOP’s formation through a multi-institutional partnership between MSPAS, SJCRH and AYUVI is testament to this. The degree of cohesion within the childhood cancer policy community is strong, though this cohesion extends less to government than in El Salvador.

“Working with the government is very difficult and it requires a lot of diplomacy, a lot of strength from the foundation so that we can maintain our budget or let us work, basically.” (S1-GUA)

The childhood cancer program in Guatemala runs largely in parallel with the public health system, with resultant implications for national policy development and system integration. In both Guatemala and El Salvador, the involvement of professional leaders in regional collaborations and international partnerships has added legitimacy and political muscle to domestic policy communities; we explore this in greater detail below.

The Philippines has witnessed progressive improvements in collaboration between and transparency within organizations involved in cancer policy and care. Champions of childhood cancer initiatives in the Philippines have recognized the importance of stakeholder engagement from across health disciplines and sectors, including both private and public sector institutions. The Community-Based Cancer Care/Control Network (CCCN), established in 1998, has stewarded the development of a network of organizations dedicated to providing quality cancer care and control in the country [43]. It promotes a multi-sectoral strategic approach to improve continuing medical education, monitoring and information resources, research, evaluation, public health and clinical management initiatives. Initially focused on adult cancers, it has now begun to incorporate childhood cancer institutions and perspectives in planning endeavors.

“These are people from different sectors of society banding together to form the cancer care program. And there are those that are just not being for particular cancer problem so they’re lobbying for funding for gynecological cancers or pediatric cancers…In terms of being able to bring that program into fruition, I think it will require both public and private [involvement]” (S1/E1-PHI).

Access to essential medicines has served as a nexus for cross-sectoral advocacy in respect of childhood cancer policy, with medical professional organizations working alongside hospital administrators and civil society groups to study inequities in drug access and lobby the government to enact pricing and financing policies that would attenuate them (PRA5-PHI, NGO1-PHI). Describing the collaboration amongst patient and professional organizations to make necessary medicines more accessible, one regulatory official reflected:

“That made me understand that patients actually better appreciate the value of tackling access from a broader perspective, from a health systems approach, rather than tinkering with something that’s easy to do but might not work” (PRA5-PHI).

In tandem with these formal and cause-specific institutional partnerships, provider networks have advanced in reach and sophistication: clinicians and surgeons treating children with cancer at tertiary centers are working to train local colleagues to recognize and appropriately respond to various cancer presentations (S5-PHI, E3-PHI)[39].

Ghana’s childhood cancer policy space has been shaped by the presence of a few key leaders and advocates, but remains a very small policy community with limited power and minimal cohesion across institutions and sectors (PRA1-GHA). Advocacy on adult cancers has resulted in the prioritization of breast, cervical and prostate cancers in the National Cancer Plan, and consequent coverage of care related to these diseases through the National Health Insurance Scheme [47]. The childhood cancer policy community has not had the resources or political voice to advocate as effectively, and political commitments in respect of childhood cancer have consequently lagged. Stakeholders noted that personnel challenges loom large in this regard: *“If we decide to sit down today and do a policy on childhood cancers*, *we probably would not have even 10 people sitting down*” (PRA3-GHA). This situation is compounded by health system governance structures in the country which exclude teaching hospitals from MOH and GHS oversight, and thereby limit channels of communication and influence for childhood cancer program leaders in national health policymaking (S2-GHA) [48].

In India, the unique governance arrangements that separate TMC from both private and government-run hospitals serve, variably, to dilute and concentrate policy community cohesion. Sheltered from the political and fiscal realities of the broader health system, TMC has been able to pursue institutional and program innovations beyond the capacity of many other centers (S3-IND). Its distinct set of rules, set in sharp relief against the complex sociopolitical backdrop of India’s health system, has arguably limited opportunities for policy community coalescence around issues of mutual concern. At the same time, TMC’s autonomy, and the successes bred by it, have positioned the institution as a national leader in the governance of childhood cancer. It has at times functioned as a lightning rod for cross-institutional engagement on policy development, political advocacy, and system reform. Its stewardship of the creation of a National Cancer Grid (NCG) is a prominent example of this (S2-IND). A network of cancer centers, civil society groups, and academic institutions, the NCG has fostered the development and national adoption of uniform clinical standards, distributed programs of specialty training and education, and augmented research infrastructure that is beginning to transform the reach and impact of childhood cancer care in India [55].

#### External actors

The role of external actors has likewise varied across countries, with differing impacts. In Ghana, international donor priorities remain principally focused on communicable diseases. World Child Cancer has invested in sustained partnerships with domestic leaders on childhood cancer advocacy and system strengthening, with particular emphasis on developing institutional networks for early diagnosis and referral through health system tiers (NGO1-GHA). However, these partnerships retain limited capacity to shape national policy formulation or budgetary priorities (NGO2-GHA). In El Salvador and Guatemala, the evolution of a Central American consortium of childhood cancer institutions, Asociacion de Hemato-Oncologia Pediatrica de Centro America (AHOPCA), engendered regional cohesion and facilitated integral support from international twinning partners, notably SJCRH [51,56].

“From the medical point of view, St. Jude’s, Dana Farber, relationships within Europe and with Canada…have helped us a lot in developing protocols and managing cases, developing diagnostic platforms, improving protocols, consultations and cases, different tumor boards with different protocols and research, publications, you name it.” (S1-GUA)

These external actors served, variously, as knowledge brokers, financiers, and political advocates for childhood cancer care in El Salvador and Guatemala, strengthening institutional capacities sufficiently position childhood cancer as an issue on national agendas for broader health system reform (S2-GUA) [51]. In the Philippines, domestic involvement in *My Child Matters*, an international multi-institutional initiative to improve the survival of children with cancer in LMIC, has spurred the development of a network of 37 health facilities to care for children outside of Manila, and has orchestrated a public awareness campaign to improve early detection of childhood cancer [57]. A comparable partnership on early detection exists between UNOP and My Child Matters in Guatemala. In India, by contrast, external actors have played a far less influential role in shaping national political priorities related to childhood cancer. Stakeholders argued that the early dominance of TMC, the presence of established government institutions and health sector strategies, and domestic capacities for professional association and civil society advocacy combined to limit the impact of external actors on childhood cancer policy in India–for better or for worse (S2-IND, PRA2-IND, NGO1-IND).

### Ideas and issue characteristics

As compared with political contexts and actor dynamics, far greater uniformity is evident in respect of the ideas and issue characteristics conditioning the relative priority of childhood cancer across the country case studies. Ideas–which encapsulate the understanding and portrayal of a given health issue, both within the policy community and to the broader public–often act as critical determinants of the political attention for it. Issue characteristics–namely, key features of the problem, including its severity, credible indicators to understand and contextualize it, and the existence of effective interventions to respond to it–are integral filters for such ideas. A dominant ideational theme attached to childhood cancer in all the countries studied is its framing as an NCD. Childhood cancer policy communities have internally recognized the need to define the problem and their prioritized responses to it in terms of wider policy currents advancing NCD care, and to paint external portrayals of it in these terms (S3-ES, PRA3-ES, S1-GUA, S3-IND, NGO2-GHA). Implicit in these formulations of the problem, and solutions thereto, is a recognition that health system strengthening arguments provide a critical link to governmental perspectives and priorities [58, 59]. Where national governments have articulated NCD priorities, as they have in the Philippines, India, El Salvador, and Ghana, childhood cancer advocates have endeavored to hitch their issue to these priorities, with varying success (PRA6-ES, NGO1-GHA, S2-PHI, NGO2-IND).

Degrees of success in this regard have been conditioned in large part by the political and power dynamics described above, but also by the characteristics of the issue itself in each context. The availability of credible indicators of the problem and its severity has been instrumental in this regard. The lack of reliable population-based data on childhood cancer incidence in the study countries–as in most LMIC–constitutes a critical barrier to enhanced political prioritization (PRA2-GHA, HI1-ES, HI3-PHI, S4-GUA, S5-IND). In political terms, childhood cancer suffers from intrinsic limitations in the size of its burden relative to other health system issues in many LMIC contexts. In Ghana, for instance, as in many other LMIC, disease burden serves as a principal determinant of health system priority-setting; this frame negatively influences perceptions of the opportunity costs of treating childhood cancer as against competing system issues (PRA3-GHA) [45]. This inherent limitation in prevalence is exacerbated by uncertainties related to quantifying the childhood cancer burden in many countries. The Philippines has developed loco-regional population-based cancer registries, but no analogous national registry; efforts are ongoing to enhance both population- and hospital-based registration, with emphasis on sentinel centers (HI2-PHI). Historically, El Salvador and Guatemala operated institutionally-based childhood cancer registries, but lacked true population-based registration. In partnership with Dana-Farber Cancer Institute in the US, both countries have recently undertaken development of population-based childhood cancer registries [60]. Ghana lacks both comprehensive institutional and population-based cancer registries; the fact that childhood cancer is not addressed in the Ghana Health Service’s annual report stymies efforts to generate priority for the collection of incidence data.

“Unfortunately, childhood cancer is not covered in the annual health report, when they are looking at the conditions that present. It’s not covered. If you don’t have the Ghana Health Service reporting on the incidence of childhood cancers, then who talks about them?” (NGO1-GHA)

India has the most extensive cancer registration program among the sample countries: it operates 28 population- and 7 hospital-based registries under the National Cancer Registry Program [61]. Even in this context, the data’s reflection of reality is compromised by incomplete population coverage and under-diagnosis (HI1-IND). The variable quality of systems of program monitoring and evaluation compounds issues with registry data by constraining appraisals of interventions that respond to the problem. Whereas hospital-level data on childhood cancer outcomes exist, in varying degrees of accuracy and specificity, across sentinel institutions in the countries studied, little system-level data exists on program implementation, the effect of specific interventions, or the cost-effectiveness of elements of childhood cancer care (PRA2-IND). Building quality repositories of such data will prove crucial to both domestic and international attempts to advance childhood cancer as a political priority in these and other LMIC.

## Discussion

### Comparative national priority of childhood cancer in health policy agendas

Our results demonstrate that the priority for childhood cancer in national health policy agendas varies considerably across the countries studied (S3 Table). Ghana has placed relatively little national priority on childhood cancer. While it has articulated both an explicit NCD strategy and a National Cancer Plan, concrete priorities and actions in respect of childhood cancer have remained elusive (PRA2-GHA, NGO2-GHA). Key health system stakeholders in Ghana–including service providers, the Ministry of Health, Ghana Health Services, and the Non-Communicable Disease Control Program, among others–have endorsed the need to strengthen childhood cancer programs and services [46, 48]. However, minimal political commitment to allocating the requisite financial resources and insurance provisions has materialized, and a lack of cohesion amongst stakeholders persists, hampering the development of policies and mechanisms of governance essential for system change (PRA1-GHA, BMA1-GHA).

Contrasting routes to the generation of priority are evident in El Salvador and Guatemala, on the one hand, and the Philippines, on the other: the mounting, through variable, presence of childhood cancer on national agendas in the former has been influenced importantly by grassroots forces; in the latter, though key grassroots initiatives exist, top-down forces have proven decisive (PRA1-ES, S1-GUA, PRA4-PHI, HI4-PHI) [33, 42]. In both El Salvador and Guatemala, actor power has played a central role in generating national priority for childhood cancer, despite political contexts less conducive to requisite health system reforms. On the force of foundational support from external twinning partnership with an HIC tertiary care institution (SJCRH), well-organized and -resourced civil society organizations have disrupted legacies of fragmented system governance and financing to create *de novo* priority for childhood cancer care (NGO3-ES, S2-GUA). In El Salvador, this has gradually induced broader political support and increasing health system integration; in Guatemala, fundamental challenges to health system adoption of innovations in pediatric cancer care persist (PRA3-ES, S1-ES, S1-GUA, S2-GUA). A paramount challenge in the Philippines remains wide disparities in health outcomes that exist across all ages and diseases, including childhood cancer [39]. However, the pediatric cancer community has recognized a window of opportunity to expand access and reduce disparities in childhood cancer care through the political prioritization of UHC and NCDs in current health system reforms (PRA4-PHI, HI4-PHI).

The example of Tata Memorial Center (TMC) in India embodies a hybrid of these paths to political priority. Its evolution from private sector philanthropic origins to privileged public embedding represents a unique trajectory towards political prioritization and health system integration (S5-IND, PRA4-IND). The role of an established and uniquely empowered private actor was instrumental in initiating change (S2-IND, PRA1-IND). Notably, this historical trajectory arguably represents a domestic manifestation of the role played by SJRCH in El Salvador and Guatemala, suggesting the import of a ‘centre-of-excellence’ in at least initiating, if not scaling, policy priority by conferring immediate political capital. Sustained political priority, however, ultimately relied upon the creation of a *sui generis* public sector governance structure that assured TMC institutional hegemony and outsized system influence (S5-IND, PRA3-IND).

Viewed in aggregate, these countries’ health system experiences suggest that political context and actor power dynamics most influence the political priority for childhood cancer in LMIC. While ideas and issue characteristics related to childhood cancer have important impact, their relative uniformity across different sociopolitical environments limits their explanatory power as determinants of policy change.

### Policy implications: Opportunities to prioritize childhood cancer on national health agendas

Comparative evidence of the determinants of prioritization of childhood cancer point to opportunities to increase its relative importance, while remaining sensitive to broader health system dynamics (Table 2). Taking careful stock of encompassing political contexts–in particular, rhetorical and policy priority placed on NCDs and UHC–provides an opportunity to place childhood cancer firmly on national health agendas. The SDGs have spurred a renewed global and national emphasis on achieving UHC–a target for SDG 3 [62,63]. In tandem, increasing political attention to the rising global burden of NCDs has placed cancer squarely on policy agendas of the World Health Organization (WHO) and many LMIC [64, 65]. The unique place of childhood cancer in both of these broader narratives presents a window of opportunity to enhance its priority on national agendas [12].

**Table 2:** Generating national political priority for childhood cancer in LMIC: Barriers and enablers.

	DETERMINANTS OF PRIORITY	BARRIERS	ENABLERS
**POLITICAL CONTEXTS**	• Policy windows • National governance structure • Economic environment	• Political instability • Weak public health systems, poor coordination across system tiers • Large private health sector, poor financial protection, cost-related access barriers	• UHC and NCD prioritization • Potential for diagonal health system strengthening • Public-private partnerships • Novel streams of financing for childhood cancer
**ACTOR POWER**	• Policy community cohesion • Leadership • Guiding institutions • Civil society mobilization	• Variable engagement of government stakeholders in childhood cancer policy communities • Service decentralization diluting influence of centers of excellence • Suboptimal coordination with child health and NCD advocates	• Tight-knit professional and advocacy communities • Dedicated and empowered professional leaders • Centralized care from signal institutions • Tendency towards strong grassroots advocacy and community/foundation support
**IDEAS**	• Internal frame • External frame	• Limited knowledge of cross-national policy experiences and enablers • Competing policy priorities and perceived opportunity costs	• High degree of internal consensus about nature of the problem and ideal solutions • Strong societal resonance of childhood cancer • Unifying cause from universalized risk
**ISSUE CHARACTERISTICS**	• Credible indicators • Severity • Effective interventions	• Lack of comprehensive registry data • Small burden of disease, competing priorities • Weak horizontal health system coordination, compromising timely diagnosis and referral • Need for intensive, expensive supportive care • Lack of guidance on efficiency of interventions geared to facility and health system tier	• Serious but circumscribed health system problem • Potential for high yield in survival from treatment • International coordination on resource-adapted treatment protocols • Growing evidence of cost-effectiveness of childhood cancer treatment\

Childhood cancer is both a dominant and remediable cause of death for children globally [6]. Effective care of children with cancer requires a well-functioning health system, premised on investments in core health system competencies, including: effective governance, financing, resource management and integrated service delivery that is equitable, efficient, effective and responsive to users [8, 66, 67]. Augmenting health system capacities for childhood cancer care can have robust positive spillover effects for health care more broadly.

The importance of key health system actors in determining the relative political priority for childhood cancer in the countries studied points to actor power as a critical enabler of prioritization in other LMIC. The presence of dedicated and empowered clinical program leaders, working within and buttressed by tight-knit advocacy communities, was a signal characteristic of successful local efforts to place childhood cancer on national health policy agendas. The role of private foundations in generating novel, earmarked streams of financing for childhood cancer care proved instrumental in bridging grassroots-led institutional innovations and the fiscal realities of public health systems.

In addition to a groundswell of professional, advocacy and philanthropic organizations dedicated to children with cancer, recent commitments by global health governance institutions have altered the international policy landscape for childhood cancer. The WHO has established formal partnerships with the International Society of Pediatric Oncology (SIOP) and Childhood Cancer International (CCI), the representative organs of childhood cancer professional and parent communities globally. Leveraging these and allied private sector collaborations, the WHO recently launched a Global Initiative for Childhood Cancer, committed to achieving a 60% global survival rate for children with cancer by 2030, through increased national prioritization and capacity-building [68]. An ongoing Lancet Oncology Commission on Sustainable Pediatric Cancer Care aims to provide an evidence-based roadmap to this end [69]. Properly harnessed, these international endeavors can provide crucial political, technical, and financial support to domestic efforts to prioritize childhood cancer in national health policies and plans.

While childhood cancer bears a number of characteristics inherently conducive to prioritization–it is a serious but circumscribed problem, there is potential for substantial improvement in health outcomes, and it is gaining prominence on the global stage–it is nevertheless one in a sea of competing priorities on LMIC health agendas. The interplay of these elements has proven a strong driver of agenda setting in the countries studied, and provides a model for prioritization in a range of others.

### Study strengths and limitations

Our study has a number of important strengths. It is, to our knowledge, the first detailed examination of childhood cancer care in health system context, with emphasis on the impact of overarching governance and financing structures. In this regard, it offers unique insights into opportunities for sustainable implementation and scale-up of childhood cancer and allied NCD programs in LMIC, in contrast to the field’s historical reliance on implementing and evaluating institutional twinning partnerships. Our study also yields important evidence of the inverse: namely, how investments in childhood cancer care, properly employed, could serve to buttress foundational capacities in health systems at different stages of development.

The in-depth, multi-country case study design sets our study apart from prior work in this field, generating robust comparative data from which to identify patterns and themes that explain varied patterns of health system priority-setting and policy development. Importantly, data collection for this study was guided by use of an expert-informed, peer-reviewed health system analytic tool tailored specifically to childhood cancer. This enabled capture of the distinguishing features and dynamics of childhood cancer care, while retaining fundamental considerations common to most health system analyses. We also benefitted from the involvement of local study collaborators with intimate knowledge and lived experience of the health care systems and practices in their respective jurisdictions. Finally, both the large overall sample and breadth of stakeholders engaged in each jurisdiction facilitated theoretical saturation from the data collected, minimizing the risk of missed or under-explored themes.

Our study also has limitations. Given the cross-sectional nature of data collection, it provides a historical snapshot of childhood cancer care and health system development, without the capacity to prospectively observe or probe program evolution. This is a common feature of comparative policy analysis; nevertheless, a dynamic perspective on health system change through successive rounds of data collection could have deepened our causal understanding of childhood cancer policy and program development. As with most qualitative work, there are limits to the generalizability of our findings: the phenomena observed are products of the particular historical, political and organizational legacies alive in each study context. We sought to surmount this particularity through extensive cross-country comparisons, varied jurisdictional frames, and a large sample size. Finally, qualitative work of this nature is inherently perspectival, as researchers inevitably bring personal values and beliefs to the research process [70]. In this specific project, both the principal investigators and local leads were drawn predominantly from the childhood cancer community; country selections were premised in part on existing collaborative relationships and the potential for access to varied health system stakeholders through local investigator contacts. We strove to diversify the tacit knowledge and worldviews informing study design and conduct through: 1) core involvement of investigators with a range of roles and expertise, including health system experts outside of childhood cancer care, in the development of our health system analytic framework; 2) conduct of interviews by study team members without prior professional connection to, or in-depth knowledge of, childhood cancer; and 3) inclusion of a range of professional and health system roles in the study sample, with an emphasis on stakeholders outside of childhood cancer program contexts.

## Conclusions

Our study demonstrates that the generation of political priority for childhood cancer in a given health system is a product of the interaction of a set of fundamental structural factors. In the countries studied, political context and actor power played the most determinant roles in shaping national health agendas. Understanding the interplay of these factors in country context is essential to health system integration of childhood cancer care and sustained improvements in access, and yields important lessons for the scale-up of comparable NCD programs.

Future efforts to advance the reach and sophistication of childhood cancer and allied NCD programs in LMIC will depend to varying degrees on the presence of empowered networks of actors working to leverage local and international political dynamics. Signal changes in global health narratives, and in the corresponding priorities of global health governance institutions and international civil society organizations, provide an unprecedented window of opportunity to this end. Progress in childhood cancer survival globally will depend not only, or even principally, on scientific advances in disease pathogenesis or treatment, but on attention to the place of childhood cancer in the shifting mix of political, social, and economic priorities in diverse human societies.

## Supporting information

S1 Table Conceptual framework for analyzing factors determining the political priority of health issues. (DOCX)

S2 Table POSIT health system stakeholder categories. (DOCX)

S3 Table Political prioritization and integration of childhood cancer in national health systems: Cross-cutting challenges and sample country solutions. (DOCX)

S1 Fig Study interview guide. (DOCX)
