# Supplementary material for: Political priority and pathways to scale-up of childhood cancer care in five nations
Source: PLoS One. 2019 Aug 19;14(8):e0221292. doi: 10.1371/journal.pone.0221292 (PMC6699697; doi:10.1371/journal.pone.0221292)
Supplement: S1 Table — (DOCX) [file pone.0221292.s001.docx]

Denburg AE et al. Political priority and pathways to scale-up of childhood cancer care in five nations

**Supporting Information**

**S1 Table.** Conceptual framework for analyzing factors determining the political priority of health issues

| Domain | Description | Key factors |
| --- | --- | --- |
| Political context | The environments in which actors operate | - Policy windows: political moments when conditions align favorably for an issue, presenting opportunities for advocates to influence decision-makers - Economic environment: the nature of resource generation and distribution for health initiatives in system context; the role of, and interactions between, public and private spheres of health care financing - Governance structure: the degree to which norms and institutions operating in a sector provide a platform for effective collective action |
| Actor power | The strength of individuals and institutions concerned with the issue | - Policy community cohesion: the degree of coalescence among the network of individuals and organizations centrally involved with the issue - Leadership: the presence of individuals capable of uniting the policy community and acknowledged as strong champions for the cause - Guiding institutions: the effectiveness of organizations or coordinating mechanisms with a mandate to lead the initiative - Civil society mobilization: the extent to which grassroots organizations have mobilized to press national and international political authorities to address the issue |
| Ideas | The ways in which those involved with the issue understand and portray it | - Internal frame: the degree to which the policy community agrees on the definition of, causes of, and solutions to the problem - External frame: public portrayals of the issue in ways that resonate with external audiences, especially the political leaders who control resources |
| Issue characteristics | Features of the problem | - Credible indicators: clear measures that show the severity of the problem and that can be used to monitor progress - Severity: the size of the burden relative to other problems, as indicated by objective measures such as mortality levels - Effective interventions: the extent to which proposed means of addressing the problem are clearly explained, cost-effective, backed by scientific evidence, simple to implement, and affordable |

Modified from Shiffman and Shah (2007)^15^
