# Supplementary material for: Political priority and pathways to scale-up of childhood cancer care in five nations
Source: PLoS One. 2019 Aug 19;14(8):e0221292. doi: 10.1371/journal.pone.0221292 (PMC6699697; doi:10.1371/journal.pone.0221292)
Supplement: S2 Table — (DOCX) [file pone.0221292.s002.docx]

**S2 Table.** POSIT health system stakeholder categories

| **Stakeholder Category** | **Examples** | **Administrative Level(s)** |
| --- | --- | --- |
| **Policy and Regulatory Authorities** | Ministry of Health | National |
|  | Department of Health | Regional, District |
| **Bilateral/Multilateral Agencies** | WHO, World Bank, United Nations | National, Regional, District |
| **NGO/Civil Society Partners** | NGOs, Faith-Based Organizations, Foundations, Human Rights Organizations | National, Regional, District, Community |
| **Service Providers** | Medical Providers, Ancillary Service Providers (Public) | National, Regional, District, Community |
|  | Medical Providers, Ancillary Service Providers (Private) | National, Regional, District, Community |
| **Patient Groups and Organizations** | Advocacy Groups, Support Groups | National, Community |
| **Health Information Organizations** | Government/Vital Statistics Agencies | National |
|  | Cancer Registry Organizations | National, Institution |
|  | Research Organizations | National, Institution |
| **Educational Organizations** | Medical Training Programs, Academic Partners | National, Regional, Institution |
