# Supplementary material for: Political priority and pathways to scale-up of childhood cancer care in five nations
Source: PLoS One. 2019 Aug 19;14(8):e0221292. doi: 10.1371/journal.pone.0221292 (PMC6699697; doi:10.1371/journal.pone.0221292)
Supplement: S3 Table — (DOCX) [file pone.0221292.s003.docx]

**S3 Table.** Political prioritization and integration of childhood cancer in national health systems: Cross-cutting challenges and sample country solutions

| Domain | Challenges | Programmatic Solutions | | | | |
| --- | --- | --- | --- | --- | --- | --- |
|  |  | **El Salvador** | **Guatemala** | **Philippines** | **India** | **Ghana** |
| Governance | *Health system and policy environment*:   - Insufficient governance capacities - Weak or fragmented public health system   *Planning and priority setting*:   - Competing health system priorities | - Integration into national pediatric hospital network and public sector infrastructure from program inception - Horizontal system strengthening as nidus for improved coordination of childhood cancer program across care continuum | - Creation of independent governance and accountability structures for private, non-for-profit childhood cancer program - Formal engagement of governmental partner (MSPAS) in policy and program development | - Incorporation of pediatric cancer into national UHC and NCD health system reforms - National cancer control plan (PCCP) broadened to include childhood cancer - Government designation of comprehensive cancer centers | - Distinct governance structures and channels of accountability for leading institution - Institutional leadership of policy community for innovation and system reform | - Presence of strong institutional leaders for policy and advocacy on childhood cancer - Centralized institutional oversight of childhood cancer care program; push for integration with system priorities |
| Financing | *Resource generation*:   - Constrained public resources - Inconstant financing flows | - Creation of public-private funding model to supplement public resources - Foundation generation of novel philanthropic funds and revenues streams - Engagement of civil society for coverage of indirect medical costs and mobilization of community donations | - Creation of public-private funding model to minimize reliance on steady government funding - Novel and sustained channels of funding generated through private foundation (AYUVI) - Early engagement of national and international philanthropic partners for financial support | - Mix of public and private resource generation - Recent creation of public ‘sin tax’ to augment DOH revenues - DOH pooling and allocation to public hospitals - Corporate donor and philanthropic foundation supplementation of specific institutional or program budgets | - TMC resources derived from mix of dedicated channel of government funds (DAE), patient-specific public and private insurance schemes, corporate donations, private philanthropy | - Majority of total health expenditure from government sources, covers elements of in-hospital services at tertiary referral centers |
|  | *Resource distribution*:   - Gaps in health coverage, limited financial risk protection | - Annual government budgetary allocation contingent on program reporting - Dedicated government provision of funding for institutional overhead and select service costs at tertiary referral hospital (HNNBB) | - Annual government budgetary allocation based on program reporting - Pooling and direct allocation of ear-marked funds by AYUVI - Free coverage of direct and majority of indirect medical costs by UNOP/AYUVI | - Means-tested government subsidies (Z Benefit) for direct medical care for patients with ALL - ‘No balance billing’ policies for indigent patients - Variable provision of philanthropic support for indirect costs of care | - Comprehensive coverage of childhood cancer care at TMC, through institutional resources and supplementary support from ImPaCCT - Wide variations in coverage within and across Indian public and private health sectors | - External donor support of childhood cancer program development - Need for enhanced coverage of childhood cancer in NHIA |
| Service delivery | *Health workforce and infrastructure*:   - Limited investment in human and infrastructural bases of childhood cancer care | - Construction of autonomous outpatient pediatric cancer center on government-donated land - Utilization of public infrastructure and human resources through partnership with national referral hospital - Foundation-supported competitive salary and professional opportunities to improve specialist retention - Development of national training programs to improve primary care provider awareness of pediatric cancer - Regional cooperative and international partnerships for specialized training, education, and research | - Construction of stand-alone hospital for pediatric cancer care - Leverage of existing public medical and social services through external contracting as needed - Participation in regionalized health workforce training for pediatric subspecialists - Regional cooperative and international partnerships for education and research - Regional standardization and evaluation of resource-adapted treatment protocols | - Early government investment in specialized workforce training, including a recognized pediatric oncology fellowship and specialty nursing training program - ALLMAP and Z benefit package allocate funds for training of allied health professionals at designated treatment sites - Establishment of national pediatric oncology professional society and participation in Western Pacific cooperative group promote standardization and evaluation of context-specific care protocols | - Robust programs of pediatric oncology specialty training across the country - Centers of excellence in childhood cancer care - NCG adoption of uniform clinical standards, distributed training programs, and cooperative research infrastructure - Recent establishment of Indian Pediatric Oncology Group (InPOG) for cooperative clinical trials in pediatric cancer | - Limited investment in infrastructure related to childhood cancer care - Regional cooperative and international partnerships for specialized training, education, and research, mainly in Africa and India |
|  | *Essential medicines and health technologies*:   - Erratic supply of essential medicines for cancer - Cost-related access barriers | - Government adoption of WHO Essential Medicines List, legislation on right to access essential medicines - Foundation purchase of non-formulary or high-cost drugs, per international guidelines - Government approval process for independent procurement of non-formulary pediatric cancer drugs | - Foundation (AYUVI) procurement of all essential medications and technologies, per WHO EMLc and international professional guidelines - UNOP audits of institutional use and oversight of supply management | - ALLMAP ring-fenced funding for chemotherapy drugs for leukemic patients; remainder fall under general Medicines Access Program - Coordinated professional-civil society advocacy for drug price reductions and enhanced coverage | - Strong domestic generic drug production supports availability and decreases prices; however, weak pharmacovigilance of drug provenance and quality | - Strong pharmacovigilance from the Ghana National Drugs Program within the Ministry of Health - Prices monitored by GNDP and MOH - 70% of generic drugs imported from India/China - National Drug Policy adapted from the WHO Essential medicines list |
| Care access & utilization | *Social determinants and access to care*:   - Treatment abandonment due to socioeconomic and cultural barriers - Diagnostic and treatment delays due to limited diagnostic capacities and weak referral pathways | - Primary care teams (ECOS) as node for early cancer detection and referral - Philanthropic (ASAPAC) support to families for indirect costs of care - Service devolvement to primary care tier for shared-care models in palliation, supportive care, survivorship | - De-concentration of outpatient services to satellite clinics - Direct relationships with referring hospitals for newly diagnosed cases - Centralized referral of pediatric cancer care for MSPAS and public sector (unclear for private sector) | - Decentralization of care to a network of accredited treatment centers to improve access - Earlier detection improved from large national public awareness campaigns - Network of designated pediatric cancer sites improved coordination and effective use of available resources (decentralized but regionalized care) | - TMC foundation (ImPaCCT) support for indirect costs of care (nutrition, accommodation, vocational training, family psychosocial services) | - Civil society support to expand primary care capacities for early recognition and referral - Philanthropic support of indirect costs of care through international civil society and foreign aid |
| Health information systems | *Surveillance and data management*:   - Lack of reliable epidemiologic and outcome data to adjudicate system performance | - Early investment in modular electronic medical record on open access platform - Integration of pediatric cancer-specific EMR into public children’s hospital - Creation of population-based pediatric cancer registry, incorporated into MOH data | - Guatemalan Pediatric Cancer Registry initiated in 2014 - Retrospective archiving into an electronic health database at UNOP - Routine use of institutional data to for quality improvement projects and future strategic planning | - Established regional population-based cancer registry (Manila/Rinzal), ongoing development of national registry - Circumscribed database created for monitoring of ALLMAP and Z benefits package recipients | - Operation of 28 population- and 7 hospital-based registries under the National Cancer Registry Program; ongoing efforts to improve data quality and expand coverage | - Institution-specific data on childhood cancer outcomes; no population-based registration or national reporting of childhood cancer incidence or outcomes |
