# Supplementary material for: Political priority and pathways to scale-up of childhood cancer care in five nations
Source: PLoS One. 2019 Aug 19;14(8):e0221292. doi: 10.1371/journal.pone.0221292 (PMC6699697; doi:10.1371/journal.pone.0221292)
Supplement: S1 Fig — (DOCX) [file pone.0221292.s004.docx]

**S1 Figure.** Study interview guide

**Study Research Questions:**

- What are the strengths and weaknesses of the existing childhood cancer program in your country, and what opportunities exist for health system and childhood cancer program strengthening?
- What are the major barriers and enablers to childhood cancer system strengthening, and to the establishment of effective universal coverage for childhood cancer services?
- How and to what degree do childhood cancer care services interface with the national health system, and what opportunities for synergy exist between childhood cancer programs and child healthcare broadly?

**Stewardship & Governance Objectives:**

1. Understand the institutional setting within which childhood cancer policies are developed. Delineate the governance and accountability structures for childhood cancer system policies and processes, including key stakeholders and outcome targets.
2. Understand the processes by which childhood cancer priorities are set within the health system and within cancer programs.
3. Understand the interface and degree of integration between childhood cancer care and a) national cancer program and policies; b) other national vertical disease programs; c) horizontal health system structures, including primary care and referral pathways; and d) childhood health programs.
4. Understand the equity issues and impacts (ethno-cultural, socioeconomic, and geographical) of current childhood cancer system practices.
5. Understand key determinants of policy agenda setting, development and implementation vis-à-vis childhood cancer in the context of the national health system and policy environment.

**Financing Objectives:**

- 1. Delineate the sources and modalities of childhood cancer care financing, and understand their relationship to broader health system financing in country.
  2. Describe models for funding and remunerating childhood cancer care services across the care continuum. Explore the impacts of such models on incentivizing behavior change, achieving system objectives (i.e., responsiveness, effectiveness, equity, efficiency) and improving system outcomes (i.e., health, financial protection, and user satisfaction).
  3. Gain insight into the political economy of health system resource generation and allocation in the country context, and its impacts upon the childhood cancer system.

INTERVIEW DATE/TIME: _____________________

**Pre-interview**

- Review purpose of study and why participant selected
- Review and collect informed consent
  - Review anonymity conditions
  - Review withdrawal rights (any point during/after conduct of interview)
- Describe nature/structure of interview

[*Interview questions and probes are intended to guide discussion towards coverage of key domains; they need not be pursued in order or exhaustively, but rather should be adapted to permit natural flow of conversation.]

*[Begin recording]*

**Introduction**

- **State date**
- **State professional role:**
  - Policy and regulatory authority representative
  - Bilateral/multilateral agency representative
  - NGO/civil society representative
  - Service provider
  - Patient group/organization representative
  - Health information organization representative
  - Educational organization representative
  - Other

***Preamble:***

- This study is one part of a larger project examining the financial, infrastructural and political investments needed to make global improvements in childhood cancer outcomes. The project seeks to better document the global burden of childhood cancer, understand the health system dimensions of childhood cancer care in varied national contexts, and cost the scale-up of effective coverage for children with cancer globally.
- The first part of this interview is focused on understanding how policies on childhood cancer are made in your country, and how programs of care for children with cancer are governed and administered.
- The second part of this interview is focused on understanding how childhood cancer care is financed in your country, how funding priorities are determined, and how funds are administered.

**GOVERNANCE**

**Guiding interview question:**

*How are policies related to childhood cancer care made and overseen in your country?*

**I. Health System and Policy Environment**

DESK REVIEW:

1. Identify the organizations and/or committees responsible for health system policy formulation and implementation.
2. Identify the organizations and/or committees responsible for national cancer policy formulation and implementation.

INTERVIEW:

1. **Legislative and Policy Environment**: Is there national legislation or policy governing cancer treatment and care in your country? Childhood cancer?
   1. Is there national legislation or policy governing pediatric palliative care and pain management in your country?
   2. Is there national legislation or policy on opioid procurement?
2. **Policy Development**: What are the key determinants of childhood cancer policy development in your country?

PROBES

*Analytic domains: ideas, institutions, interests*

- 1. Can you describe the key institutions, stakeholders and organizational processes relevant to child cancer policy development? How do these structures and processes relate to overarching processes for health system policy and cancer policy development, respectively?
     1. Please describe the main institutional and procedural structures for health system policy formulation.
     2. Please describe the main institutional and procedural structures responsible for cancer policy formulation.
     3. Please list the key stakeholder groups involved in the development of childhood cancer related policies and programs for your country.
     4. Are there any distinct institutions or processes dedicated to childhood cancer policy development?
  2. What challenges exist in developing national policies or strategies on childhood cancer care?

1. **Cross-sectoral Policy and Program Interface**: How do existing policies and programs for childhood cancer interface with those for:

(a) the adult cancer system

(b) flagship vertical programs

(c) child health programs, and

(d) primary care and referral pathways?

PROBES

*Analytic domains: ideas, institutions, interests*

- 1. What are the key areas of integration and divergence?
  2. What strengths and challenges do these points of interface create?
  3. What opportunities exist to strengthen childhood cancer care through attention to policy and program interface across these domains of the health system?

**II. Planning and Priority Setting**

DESK REVIEW:

1. Identify existing national cancer control plans (general and child-specific) and non-communicable disease plans.

INTERVIEW:

1. **Agenda setting**: How is the government’s childhood cancer policy agenda set?

PROBES

*Analytic domains: problems, policies, politics 🡪 windows of opportunity*

- 1. How are health system priorities determined in your country? What strengths or challenges do you perceive in existing priority setting processes?
  2. How, if at all, do processes for health system priority setting influence childhood cancer policies and priorities?
  3. How do priority setting processes within the existing cancer system influence child-specific priorities and plans?
  4. Are there any distinct mechanisms for setting system priorities for childhood cancer?
  5. What factors have either elevated or diminished childhood cancer on the government’s agenda?

1. **National Childhood Cancer Plans**: Does your country have a national non-communicable disease (NCD) and/or national cancer control plan (NCCP)? If yes and in public domain, please provide.

PROBES

*Analytic domains: ideas, institutions, interests*

- 1. If an NCCP exists:
     1. What prompted the development of your country’s NCCP?
     2. What are the strengths and weaknesses of the NCCP with respect to childhood cancer?
     3. Does your NCCP set any health outcome targets for pediatric cancer control? For palliative care? What outcome-based indicators are used? How are these data collected?
     4. What are the key barriers and enablers to enhancement of childhood cancer provisions and/or targets in the NCCP?
  2. If an NCCP does not exist:
     1. Considering your country’s current political and economic climates, are there any broad limitations to the development and implementation of a national cancer plan?
     2. What specific factors, if any, have prevented or forestalled NCCP development in your country?
     3. Is there a distinct strategy, plan or set of policies for childhood cancer control in the sub-national levels (i.e. provincial or district level plans, municipalities/cities etc.)?

**III. Accountability and Clinical Standards**

DESK REVIEW:

1. Identify existing national or institutional clinical and/or health workforce standards related to childhood cancer care delivery.

INTERVIEW:

1. **Implementation**: How are childhood cancer policies implemented and overseen?

PROBES

*Analytic domains: ideas, institutions, interests*

- 1. Who is accountable for the implementation of policies affecting children with cancer?
     1. Please describe any oversight processes in place for ensuring that resources are distributed and utilized as intended.
     2. Are there any ways in which the childhood cancer system is negatively impacted by misuse or misdirection of resources?
  2. What reporting and performance management structures exist in relation to policies and systems for childhood cancer care?
     1. How do these relate to accountability structures for the broader cancer system?
     2. How do they relate to accountability structures for the overarching health system?
  3. What challenges exist to childhood cancer policy implementation and oversight?

1. **Clinical standards**:
   1. Are there national- or facility-level standards of care for the treatment of childhood cancer? Please describe.
      1. Do you have specific protocols for curative treatment?
      2. Do you have specific protocols or guidelines for supportive care?
      3. Are these standards of care in the form of clinical guidelines (recommendations), protocols or SOPs (best practice standards) and/or research protocols?
      4. Do the guidelines describe the expected management at different levels within the healthcare system (e.g. clinic, smaller hospital, referral hospital etc.)?
      5. Are these standards/guidelines developed or assessed with consideration of any resource constraints that may apply within your country?
      6. Are there different protocols based on the relative high or low risk status of a child’s cancer?
      7. To what extent is research and current evidence utilized and integrated into these standards and guidelines?
      8. Are there any systems to monitor and evaluate adherence to national standards? Are there any evaluative/quality assurance components implemented with these standards/guidelines?
2. **Accreditation**:
   1. Is there an accreditation process for centres providing childhood cancer treatment?
      1. If yes, please describe: who accredits, whether there are levels of accreditation, main criteria for accreditation (e.g. patient volume, infrastructure, reporting ability), duration of accreditation. If no, please describe whether there is another form of evaluation or certification.
      2. Do all centres/facilities providing childhood cancer treatment follow the same accreditation process and standards? Please describe.
   2. What are the incentives for an institution to become accredited?
   3. What are the consequences for an institution not becoming accredited?
   4. Is there a system of accreditation/qualification of cancer treatment professionals in your country?
      1. If yes, please describe: who accredits, whether there are levels of accreditation, main criteria for accreditation, duration of accreditation. If no, please describe whether there is another form of evaluation or certification.
   5. How, if at all, have the accreditation systems (for cancer treatment centres and cancer treatment professionals) affected overall performance management within the health system?

**FINANCING**

**I. Resource Generation**

DESK REVIEW:
1. Identify the existing structures/systems for generating and/or pooling funds for the health sector and the sources of funds.
2. Please describe the existing structures/systems for generating and/or pooling funds for the health sector and the sources of these funds.

INTERVIEW:

How is childhood cancer care financed in your country (i.e. where do the funds come from and how are they generated)?

| **Source** | **Mechanism** | **Proportion** |
| --- | --- | --- |
| Government | General taxation  Payroll  Earmarked |  |
| Civil society | Charitable donation |  |
| International donor | Government ODA  Twinning institution  International institution  Innovative financing |  |
| Private | OOP  User fees  Medical savings accounts  Private insurance  Employer-based insurance |  |

What are the implications of the current financing mix/model for:

1. Resource sustainability for childhood cancer care care?
2. Priority setting and resource allocation for childhood cancer programs and services?
3. Equity of access to childhood cancer care?

How does the financing for childhood cancer care differ from that for cancer care and control in adults?

**II. Resource Distribution (Funding)**

**Please describe the existing systems of governance/organization for pooling and distributing funds for:**

- The health system
- Cancer care
- National Cancer Control Plan (NCCP) directed programs, if a NCCP exists
- Childhood cancer care

PROBE:

- What, if anything, could be improved in terms of pooling and distributing of funds?

**How are resources allocated within the national health system?**

PROBES:

- General health priorities? Disease priorities? Target groups? Target regions? Level of care (primary/secondary/tertiary)? Types of care (preventive/early diagnosis/curative treatment/palliative care)?
- Is there a formula used for resource allocation?
- How are cancer program budgets used, if at all, at the national, regional, district and/or community levels?
- *If there is a NCCP:* How has the NCCP influenced: the structures/systems for pooling and distributing of funds?
- What, if anything, would you change about how resources are allocated for the national health system?

**What is the budget allocation for childhood cancer in your country? How much is actually spent on childhood cancer care ?**

PROBES:

- Absolute amount per year and the proportion from total health expenditure indicating the currency and year of estimation
- If a national cancer center exists, its annual operating budget and proportion spent on childhood cancer

**Please describe the main modalities and streams of funding for the childhood cancer care continuum in relation to health system components (primary care, diagnosis and referral, tertiary care, palliative care, survivorship care).**

PROBES:

- Are designated bundles of resources allotted to specific childhood cancer diagnoses? Or are system components (e.g. hospital services, physician services, medicines, etc) funded through distinct mechanisms? Or is it a mix of the above? Please describe.
- Is there funding in your country to cover non-treatment related costs for cancer such as travel, food, and/or accommodation for families?
- What components of medical care are patients/families required to self-fund? What out of pocket costs are typically incurred by patients/families?
- If so, specify who provides these funds and the proportion of cost/amounts.

| **Service** | | **Source(s)** | **Mechanism** | **Proportion** |
| --- | --- | --- | --- | --- |
| Hospital services (in-patient bed, DI, surgery, pathology, radiation, supportive care) | | Government |  |  |
|  |  | NGO |  |  |
|  |  | Employer |  |  |
|  |  | PHI |  |  |
|  |  | OOP |  |  |
| Physician services | |  |  |  |
| Allied health services | |  |  |  |
| Medicines | Chemotherapy |  |  |  |
|  | Supportive |  |  |  |
|  | Opioids |  |  |  |
| Out-pt/community diagnostic services (lab, DI, biopsy, pathology) | |  |  |  |
| Ancillary/indirect costs (travel, accommodation, food) | |  |  |  |

**Please describe how the following receive payment:**

Health care institutions in general?

NCCP supported programs?

Cancer treatment centers?

PROBE:

- Does the payment mechanism for programs and institutions encourage or discourage productivity in any way? Honesty and quality assurance? Improved patient outcomes?

**Please describe how health care providers are paid.**

PROBES:

- block budgets, fee for service, salary top-ups, other incentives.
- Does this differ among doctors, nurses, lab/imaging technicians, pharmacists?
- Does this differ for NCCP-supported programs?
- What are the impacts of existing provider payment mechanisms on access to and quality of childhood cancer care?

**Is the distribution of funds to health care centers and health care providers contingent on the achievement of particular standards of care/accreditation requirements?**

PROBES:

- Are there any financial incentives for diagnosis? For decreasing abandonment? For a certain level of toxic deaths?
- Are there incentive programs to increase quality, efficiency, and accessibility of health services for cancer care? For childhood cancer care?

**Please describe the scope of health insurance coverage for cancer care and childhood cancer care?**

PROBES:

- Are all childhood cancers covered by public funding or is coverage limited to only specific childhood cancers, certain populations or certain age groups of children/adolescents with cancer?
- If funding is limited to specific cancers or populations, which ones and why were those ones selected (cost effectiveness, level of supportive care necessary, achievable cure rates, etc.)?
- Is public coverage limited to specific sites of care (i.e. only government hospitals) or is there also partial payment of private care facilities)?

*[Stop recording]*

**Post-interview**

- Thank participant
- Reiterate anonymity provisions
- Request consent to follow up with participant at later date for member checking, as needed
